# The incredible years therapeutic dinosaur programme to build social and emotional competence in welsh primary schools: study protocol for a randomised controlled trial

**DOI:** 10.1186/1745-6215-12-39

**Published:** 2011-02-11

**Authors:** Tracey Bywater, Judy Hutchings, Christopher Whitaker, Ceri Evans, Laura Parry

**Affiliations:** 1 School of Psychology, Brigantia Building, Bangor University, Bangor, LL57 2AS, Gwynedd; 2 Incredible Years Cymru, Nantlle Building, Normal Site, Bangor University, Bangor, LL57 2PZ, Gwynedd; 3 The North Wales Organisation for Randomised Trials (NWORTH), Y Wern, Normal Site, Bangor University, Bangor, LL57 2PZ, Gwynedd

## Abstract

**Background:**

School interventions such as the Incredible Years *Classroom *Dinosaur Programme targets pupil behaviour across whole classrooms, yet for some children a more intense approach is needed. The Incredible Years *Therapeutic *Dinosaur Programme is effective for clinically referred children by enhancing social, problem-solving skills, and peer relationship-building skills when delivered in a clinical setting in small groups.

The aim of this trial is to evaluate the effectiveness of the Therapeutic Programme, delivered with small groups of children at high-risk of developing conduct disorder, delivered in schools already implementing the Classroom Programme.

**Methods/Design:**

This is a pragmatic, parallel, randomised controlled trial.

Two hundred and forty children (aged 4-8 years) rated by their teacher as above the 'borderline cut-off' for concern on the Strengths and Difficulties Questionnaire, and their parents, will be recruited.

Randomisation is by individual within blocks (schools); 1:1 ratio, intervention to waiting list control.

Twenty schools will participate in two phases. Two teachers per school will deliver the programme to six intervention children for 2-hours/week for 18 weeks between baseline and first follow-up. The control children will receive the intervention after first follow up.

Phase 1 comprises three data collection points - baseline and two follow-ups eight months apart. Phase 2 includes baseline and first follow-up.

The Therapeutic Programme includes elements on; Learning school rules; understanding, identifying, and articulating feelings; problem solving; anger management; how to be friendly; how to do your best in school.

Primary outcomes are; change in child social, emotional and behavioural difficulties. Secondary outcomes are; teacher and parent mental wellbeing, child academic attainment, child and teacher school attendance. Intervention delivery will be assessed for fidelity.

Intention to treat analyses will be conducted. ANCOVA, effect sizes, mediator and moderator analyses will be applied to establish differences between conditions, and for whom the intervention works best for and why.

**Discussion:**

This trial will provide information on the delivery and effectiveness of a child centred, school-based intervention delivered in small groups of children, at risk of developing more severe conduct problems. The effects on child behaviour in school and home environments, academic attainment, peer interactions, parent and teacher mental health will be assessed.

**Trial Registration:**

UK Clinical Research Network UKCRNID8615

Current Controlled Trials ISRCTN96803379

## Background

Social and emotional wellbeing can be defined as being happy and confident, autonomous, attentive, resilient, and possessing the ability to foster good relationships and solve problems. Barriers to wellbeing are depression and antisocial behaviour [1]. Children who display early signs of anti-social behaviour and conduct problems are at high risk of early onset conduct disorder (CD). CD constitutes the largest single group of psychiatric disorders in children and adolescents and is the main reason for referral to Child and Adolescent Mental Health Services (CAMHS) [2]. Children with early onset problems are at high risk of low school attendance, educational underachievement, school dropout [3] and subsequent mental health problems [4].

Living in poverty in poor quality housing, having young and/or single parents, living with adults with unemployment or mental health problems increases the probability that children will arrive at school with social and emotional skill deficits [5]. Additional risk factors associated with higher prevalence for CD include, frequent changes of parental figures, parental psychopathology, parental substance abuse, marital problems, and poor parenting skills [6].

The Incredible Years (IY) series developed over the last 30 years, comprises a suite of programmes, for parents, children and teachers, with strong evidence of effectiveness both as treatment programmes for children with CD and as early preventive programmes (for more information on programmes and research see: http://www.incredibleyears.com). IY was one of the eleven programmes deemed a 'blueprint' programme for violence prevention following a rigorous review of 600 interventions [7]. The Incredible Years Series has a strong evidence base in Wales and is acceptable to families and schools [8-10].

Parenting programmes are very effective in developing children's social skills and reducing child behavioural problems [4,8,10-13] whilst reducing later burden on social, health, education service use and associated costs and the costs of lifetime unemployment and welfare dependence [14-17].

Limitations of parenting programmes are that improvements at home do not always transfer to school settings [18,19] with programme provision limited to the few families that can, or are willing to, access programmes. Schools are therefore dealing with significant numbers of children with behavioural and self-regulatory difficulties particularly in disadvantaged areas where levels of CD reach 35% [2,20].

In Wales initial steps to tackle the increasing numbers of children arriving in school with social, emotional and behavioural difficulties have been taken; there has been a change in the curriculum of early years education and a commitment to improve home-school partnerships [21]. Gwynedd Education Service in North Wales has implemented and established the universal IY Classroom Dinosaur curriculum in all of their 102 primary schools; however high-risk children need additional input to meet their needs. The IY Therapeutic Dina Programme [22] has been successful in increasing social and emotional competence in high-risk children by increasing social and problem-solving skills, and by strengthening peer relationships in clinical settings [23] and in a small pilot study in Wales [9].

The IY Therapeutic Programme would be ideally placed within schools, where children who would benefit form the programme could be easily identified by teachers. Teachers are best placed to deliver the programme to the children in schools, and to encourage the completion of the programme's homework tasks by children with their parent/s. School-to-home links will be forged through discussion of homework tasks.

### Summary of background literature

• Fostering social and emotional competency can reduce or prevent CD, which is prevalent in disadvantaged areas such as North Wales

• Parent programmes are beneficial but more support is needed for children in school, as behaviour 'rules' learnt in the home may not generalise to school

• Schools are an ideal arena for targeting difficult child behaviour and increasing positive behaviour whilst strengthening school-home links

• All 102 primary schools in Gwynedd implement IY programmes (Teacher Classroom Management and Classroom Dinosaur)

• The intensive IY Therapeutic Programme with high-risk children in schools may benefit children for whom the IY Classroom Dinosaur is insufficient

### The rationale for the trial

The aim of this paper is to present the research protocol for the randomised controlled trial (RCT) designed to primarily establish whether the IY Therapeutic Dinosaur School Programme, when delivered as a school-based targeted intervention, improves 'at risk' children's social, emotional and behavioural competencies compared with a waiting list control condition.

#### Secondary questions include

1. For which children is the intervention most effective?

2. What are the environmental/contextual circumstances that improve the likelihood of success?

3. Does behaviour change in school generalise to the home?

4. Does the duration of time participating in an intervention affect the likelihood of success, that is, is there a dosage affect?

5. Can the intervention be implemented efficiently and effectively with fidelity by teachers in mainstream schools?

6. Does the mental health of teachers and parents of the intervention children improve?

## Method and design

This is a 3-year study, beginning in 2010, set in primary schools in Gwynedd, North Wales. It comprises two phases; half of the proposed 20 schools will deliver the programme to the (maximum) six children allocated to intervention condition in Phase 1 (2011), with the remaining schools delivering the intervention in Phase 2 (2012). Each school will deliver the programme (if effective) to the waiting-list control children in the subsequent school year.

Phase 1 comprises three data collection points - baseline and two follow-up points at 8-month intervals. Phase 2 comprises baseline and first follow-up only due to timetabling restrictions (see Additional File 1).

### Participants

Twenty schools will be identified and recruited in conjunction with Gwynedd Education Services.

In total 240 children will participate (120 intervention, 120 control) across the 20 schools. The 240 children, aged 4-8 years will be identified as scoring at least borderline for concern on the total difficulties score of the Teacher Strengths and Difficulties Questionnaire [24].

The primary caretaker or parent (n = 240) will complete measures on themselves and their child.

Teachers with a participating child in their class will complete a brief demographic form and a measure of teacher stress; Teacher sample size will be determined after recruitment of families.

Forty facilitators (teachers, or teachers partnered with classroom assistants), two from each school, will deliver the intervention to the children. The facilitators will be chosen by the school and will have undertaken relevant IY training to enable delivery.

Participant summary:

• 20 schools (10 in each phase)

• 240 children, 12 in each school, 6 intervention and 6 waiting list control

• 240 parents (primary caretaker of child participants)

• Teachers (of participant children, n = TBC following recruitment of families)

• 40 facilitators (teachers and classroom assistants)

See Additional File 2 for the Consort diagram of flow of participants.

### Recruitment

Research staff will score the Teacher SDQs and inform schools of the initials and dates of birth of the highest scoring eligible children. School staff will initially approach the parents. If interested parents will sign a 'note of interest' form to include contact details and forward to the research team. The research team will telephone the parents to arrange a home visit to discuss the research and recruit the families.

Parents will consent for themselves and on behalf of their child. Consent will be sought at the initial home visit, which will be conducted in the parent's preferred language of Welsh or English. The information and consent forms are available in Welsh and English. The parent will have a week, if required, to decide whether to participate and will be encouraged to discuss participation with their partner, family and friends.

The information sheet also makes explicit that participants, having given their consent, will be free to withdraw their child and themselves from the study at any time without affecting future access to family or children's services. Confidentiality will be assured, unless the researcher has cause for concern regarding child protection issues.

Parents will be informed that their child will be chosen randomly as to whether they receive the intervention in the first or second year of delivery (intervention or waiting list control respectively).

### Inclusion Criteria

• The index child will be rated by their teacher as borderline for concern or above on the screening measure - Teacher Strengths & Difficulties Questionnaire [24]

• The child will be 4-8 years of age

• The child and parent speak fluent Welsh and/or English

• The parent understands the information given and consents to:

◦ Their child attending the programme

◦ Their child being observed

◦ Their child completing a problem-solving task and being audio-recorded

◦ Completing questionnaires about themselves and their child

◦ Their child being randomly allocated

◦ The programme delivery being filmed for facilitators' supervisory meetings and to establish level of fidelity in delivery

◦ Their child's school academic and attendance records being accessed

### Exclusion criteria

Other than the negative of the inclusion criteria no additional exclusion criteria are imposed.

### Intervention

The IY Therapeutic Dinosaur Programme [22] is an 18-week (2 hours/week) child-training curriculum that strengthens children's social, emotional, behavioural & academic competencies. It is delivered to groups of up to six children.

It is part of the successful evidenced based IY Series, a suite of programmes which include parent, child and teacher programmes. All IY programmes are manualised and require trained facilitators.

The programme will be delivered during school time as part of the typical school day. School staff will receive training prior to programme delivery, and will receive supervision throughout delivery to ensure that the programme is delivered as it was developed.

The programme promotes understanding & communicating of feelings, problem-solving strategies, managing anger & friendships. Puppets, role-play, and video clips are used to make it a fun learning experience.

The Dinosaur School programme sessions cover six separate programmes:

1. Making new friends and learning school rules;

2. Dina Dinosaur teaches how to do your best in school;

3. Understanding and detecting feelings;

4. Detective Wally teaches problem-solving steps (including anger management);

5. Molly Manners teaches how to be friendly;

6. Molly explains how to talk to friends.

Wally, Molly and Dina Dinosaur are puppets. Children relate better to puppets than to therapists and are more likely to imitate their appropriate behaviour [23]. Each session includes activities such as "feelings" and "let's suppose" games, co-operative art projects and guessing games to improve co-operation skills. Group leaders praise and reward appropriate behaviours by labelling the behaviour and awarding "dinosaur chips". Weekly homework activities involve the child talking to their parent/s about what they have learned to encourage positive parent-child interaction.

For more programme information see http://www.incredibleyears.com

### Facilitators

The IY Teacher Classroom Management training and the IY Classroom Dina curriculum training has been undertaken by staff in all of the proposed schools. All facilitators will undergo the additional IY Therapeutic Dina training prior to delivery. The 40 facilitators will be teachers and/or classroom assistants working in the participating schools.

During intervention delivery facilitators will attend supervision meetings led by an IY trainer (the second author) once per month during the 18 weeks, for delivery support and to ensure implementation fidelity. Clips from their taped sessions will be played to facilitate discussion and feedback.

School-based delivery support will be provided by a teacher seconded to IY Cymru for the duration of the intervention delivery.

### Measures

Measures will be completed by teachers, parents, children and facilitators and collected by the research team. Demographic questionnaires will be completed by parents and teachers at baseline only. Primary and secondary measures will be administered at all time points.

*The primary outcome measure *is the Teacher SDQ, also used here as the screening measure. It is a 25-item inventory to assess the occurrence of particular behaviours that have been associated with conduct problems, hyperactivity, emotional symptoms, peer problems and pro-social behaviour in children aged 4-16. A high total difficulties score denotes higher occurrence of problematic behaviours [24].

#### Secondary outcome measures

For children:

• The Eyberg Child Behaviour Inventory across all time points is a 36-item inventory, completed by the parent for the assessment of frequency and intensity of behavioural problems in children aged 2-16 years [25]. A high score in each of the subscales denotes more frequent/intense problem behaviour.

• The Bangor Dinosaur School Questionnaire has been developed by Professor Hutchings [26], to assess levels of social and emotional competence. A high score denotes higher rates of social/emotional competence.

• The Wally Problem Solving test assesses children's problem-solving skills and solution types (positive and negative) in response to hypothetical problem situations http://www.incredibleyears.com.

• Blind observation of peer interaction to assess friendship skills (observation tool under development)

• Child interview to ascertain number of friends a child has

• Academic attainment - assessed by school records

• School attendance - assessed by school records

For parents (self-reports):

• The Parenting Scale questionnaire [27] is a 30-item inventory is designed to measure dysfunctional discipline practices in parents of children aged 1.5 to 4 years, but has been used successfully in older children up to age sixteen [8]. A high score denotes high rates of dysfunctional practices.

• The Beck Depression Inventory [28] is a 21-item measure to assess the severity of characteristic attitudes and symptoms associated with depression. A high score relates to increased levels of depression.

• The Warwick-Edinburgh Mental Well-being Scale (WEMWBS) [29] is a 14-item positively worded measure assessing mental health. The higher the score the higher the levels of well-being.

For teachers (self-report):

• The Teacher Stress Inventory (TSI) [30] is a 20-item measure to examine the effect of difficult classes, impolite pupils and maintaining class discipline on teacher stress levels. The higher the score the higher the stress levels.

Additional measures relating to programme delivery and child responsiveness consist of:

• Implementation fidelity checks - facilitator-completed after each week's session has been delivered

• Child Dina homework completion rates - facilitator assessed

• Attendance levels of child at Dina group - facilitator assessed

### Data Collection

Parent measures will be completed during home visits by the research team. A £10 cash incentive will be paid to the parent at the end of each data collection visit on completion of measures.

Teacher measures will be completed independently during school time and collected by researchers.

Child data will be collected by researchers during school time, by prior arrangement with the schools.

Research staff will be trained in the observational tool until 70% inter-rater reliability is obtained. At least 20% of 'blind' observations will occur with two researchers to gauge inter-rater reliability. Frequent practice and trouble-shooting meetings will be held to maintain reliability levels.

Facilitator implementation checklists and child programme attendance will be collected after each weekly Dina session.

Teacher and child attendance levels will be collected for each term. Child academic attainment, by means of expected and achieved grades, will be collected at each time point from Gwynedd Education Central database, or requested through individual schools.

### Sample size and power calculation

The programme will be delivered to a maximum of six children within a group format. To account for any potential difficulties in recruitment or attrition the power calculation was conducted based on 4 children per condition per school, i.e. N = 160, 33% below the agreed target of 240.

A factorial design with two factors of condition and school with two and twenty levels has forty cells (treatment combinations). A total of 160 participants are required to provide 4 participants per cell. This design achieves 88% power at a 5% significance level.

### Randomisation

This is a pragmatic, parallel, RCT, with children individually randomised on a 1:1 basis, intervention or waiting-list control.

Individual (child) block randomisation within school will be applied with gender as the stratification variable. The North Wales Organisation for Randomised Trials (NWORTH), an independent registered trials unit, will perform the randomisation process using a computer programme written specifically for this trial. Randomisation will occur after eligibility has been established, informed consent obtained and baseline measures collected from parents.

### Blinding

Randomisation will be conducted by NWORTH, with results of allocation forwarded to the Principal Investigator; all other researchers will be blind to condition. Parents, children and teachers will not be blind. Participants (parents and children) will be respectfully asked not to discuss the intervention with the researchers during follow-up interviews and collection of the research measures.

### Statistical analyses

Characteristics of the sample (child and parent) will be analysed and differences (if any) between the two conditions, intervention and control (and lost participants), will be recorded.

Demographic variables (e.g. parental status, ethnicity, education and income levels) will inform the mediator and moderator analysis - for whom the intervention worked best and why.

The main analysis is intention to treat (ITT), to include all participants regardless of protocol deviations and participant compliance or withdrawal, performed according to the assigned treatment group.

In addition to the ITT analysis a per protocol analysis will be conducted on data from participants who have remained in the study, attended at least one intervention session and have completed measures at each time point.

Different methods of dealing with missing data will be explored following data collection and establishment of how many and what type of missing values are achieved.

The difference between the intervention and control conditions on follow-up scores will be based on a mixed model analysis of covariance (ANCOVA), run on SPSS, of the response taking account of the random school effect, fixed treatment effect, gender, age/school year, and the baseline response value. Any similarities within the intervention group, due to participating in a group format intervention, will be controlled for in the model.

Effect sizes will be calculated using Cohen's (1988)[31] guidelines.

#### Statistical summary

Statistical analyses, including subgroup analyses and adjusted analyses, indicating those pre-specified and those exploratory will be conducted to include (amongst others):

• Sample characteristics

• ANCOVA will establish differences between conditions & across time points

• Effect sizes will be calculated using Cohen's d

• Mediator & moderator analyses will establish whom the intervention works best and why

• Regression analyses & correlations will assess scores on a measure predicting, or having a relationship, with another

## Discussion

This trial will provide important information on the delivery and effectiveness of a child centred intervention delivered in small groups of children, at risk of developing more severe conduct problems, in school settings by school staff. The effects on child behaviour in school and home environments, academic attainment, peer interactions, parent and teacher mental health, will be assessed.

A number of challenges exist in carrying out the proposed study; the biggest challenge is that of parental engagement and consent. The parents will initially learn of the trial through school staff. It is important that school staff are fully aware of the details of the study and are fully briefed with the most effective means of presenting the trial to parents in the most positive light to ensure that parents do not feel stigmatised, or in any way to blame for their child's social, emotional, or behavioural difficulties. Individual meetings with the Principal Investigator, Head Teachers and proposed facilitators will serve to brief school staff and alleviate any concerns on engaging with parents. Staff will be given information sheets and a template letter, summarising the study, to give to parents to aid discussion. Parents will mainly be approached on a one to one basis during parents' evening at school. School staff will help parents complete a 'note of interest' form with contact details and child details to be sent to the research staff to enable initial contact to be made. Parents will be encouraged to contact the research team or school staff with any queries.

Teachers will receive training to deliver the programme as part of the trial. Teachers will be required to take video recordings of their session delivery to monthly group supervision, undertaken by an IY Mentor. To overcome possible reticence of showing the recordings teachers will choose which sections of the recording to show at supervision for discussion, for example a section of the session that they were proud of, or a section of a session that they felt could have gone better to receive guidance and feedback on.

Despite these, and other, possible challenges the strength of the trial lies in the fact that three main elements when evaluating a complex intervention, as outlined by the MRC will be included, namely the assessment of process, implementation and outcomes [32].

### Ethics and research governance

The trial is conducted in compliance with the principles of the Declaration of Helsinki (1996) and the principles of Good Clinical Practice (GCP).

The study protocol and other documentation have been approved by North Wales Research Ethics Committee (REC), and School of Psychology, Bangor University REC. Any subsequent protocol amendments will be submitted to the RECs for approval.

Both RECs will be provided with progress reports and a final study report The Charity and University's lone worker, child protection and safeguarding policies will be adhered to.

### Data management, protection and confidentiality

Data will be handled in accordance with the Data Protection Act (1998). Databases with personal, identifiable information will be password protected on multiple levels and stored on a secure server. Statistical databases will hold non-identifiable data. All questionnaires will be inputted at the item level.

Twenty percent of inputted data will be checked to assess input error rates. Participant identifiable data is to be shared only within the research team on a need-to-know basis.

Confidentiality will be maintained and no one outside the trial team will have access to the database. The completed non-identifiable questionnaires will be stored in a locked cabinet at the University.

The trial database is securely held and maintained on the University's research data protection server, which is regularly backed up.

### Publication

The trial results will be written up for submission to a peer reviewed journal. No data relating to individuals will be identified in any publication.

## Competing interests

All authors, with the exception of Professor Judy Hutchings, declare no competing interests. Professor Judy Hutchings provides training to school and service staff in all IY programmes and is the Director of Incredible Years Wales and the Director of the Charity that holds the grant. Professor Hutchings is answerable to the Charity trustees.

## Funding

This project is funded by the BIG LOTTERY: C1119A1193. Incredible Years Cymru is the grant holder, with Bangor University and Gwynedd Education as partners. The funders are not involved in the design of the study, in the collection, analysis, or interpretation of data. The funders were not involved in the writing of the manuscript for publication.

## Supplementary Material

Additional file 1 **Gannt Chart**. A chart to show timeline of trial. Click here for file

Additional file 2 **Participant flow chart**. A chart to show flow of participants through the trial. Click here for file
