# Supplementary material for: The incredible years therapeutic dinosaur programme to build social and emotional competence in welsh primary schools: study protocol for a randomised controlled trial
Source: Trials. 2011 Feb 11;12:39. doi: 10.1186/1745-6215-12-39 (PMC3044096; doi:10.1186/1745-6215-12-39)
Supplement: Additional file 1 — Gannt Chart. A chart to show timeline of trial. [file 1745-6215-12-39-S1.PDF]

Additional file 1

Lottery funded project: Evaluating the IY Small Group Dina in Gwynedd

Activity for:	April-June 2010	July-Sept 2010	Oct-Dec 2010	Jan-Mar 2011	April-June 2011	July-Sept 2011	Oct-Dec 2011	Jan-Mar 2012	April-June 2012	July-Sept 2012	Oct-Dec 2012	Jan-May 2013
**Gwynedd Education (Phase 1)**	Orientation meeting (May 4th)	*Quarterly Steering group June 18th – key members	Attend 2-day training, 2 staff/school Identify eligible children	Deliver programme Jan-May 18 wks, 6 intervention children per school<br><br>Attend supervision			Deliver programme 18 wks, 6 control children per school					
**Gwynedd Education (Phase 2)**					Orientation meeting		Attend 2-day training, 2 staff/school Identify eligible children	Deliver programme Jan-May 18 wks, 6 intervention children per school<br><br>Attend supervision			Deliver programme 18 wks, 6 control children per school	
**IYW Centre**	Orientation meeting (May 4th)	*Quarterly Steering group June 18th – key members		1 day/month supervision at Bangor University for Phase 1 delivery			1 day/month supervision at Bangor University for Phase 2 delivery					
**IY Cymru (seconded teacher)**	Orientation meeting (May 4th)	*Quarterly Steering group June 18th – key members	Phase 1 schools, 2 visits to present project & answer queries	Teacher regular support visits to schools		Phase 2 schools, 2 visits to present project & answer queries	Teacher regular support visits to schools					
**Research Team**	Orientation meeting (May 4th)<br><br>Recruit staff<br><br>Ethical approval	*Quarterly Steering group June 18th – key members	Phase 1 baseline data collection – home & school, Sept-Dec<br><br>Randomise Phase 1		Phase 1, follow-up 1 data collection – home & school June-Sept<br><br>Phase 2, baseline data collection home & school, Sept - Dec Randomise Phase 2			Phase 1, follow-up 2 data collection – home & school	Phase 2 follow-up 1 data collection – home & school, May onwards		Final data analyses<br><br>Final written reports**<br><br>Conference presentations<br><br>Publication in peer reviewed journals	

*Dates of future steering groups to be confirmed at this meeting. Progress reports will be presented at each meeting
**Interim/yearly reports will be submitted to funders in addition to the final report
