# Supplementary figures and images for: The incredible years therapeutic dinosaur programme to build social and emotional competence in welsh primary schools: study protocol for a randomised controlled trial
Source: Trials. 2011 Feb 11;12:39. doi: 10.1186/1745-6215-12-39 (PMC3044096; doi:10.1186/1745-6215-12-39)

## Additional file 2

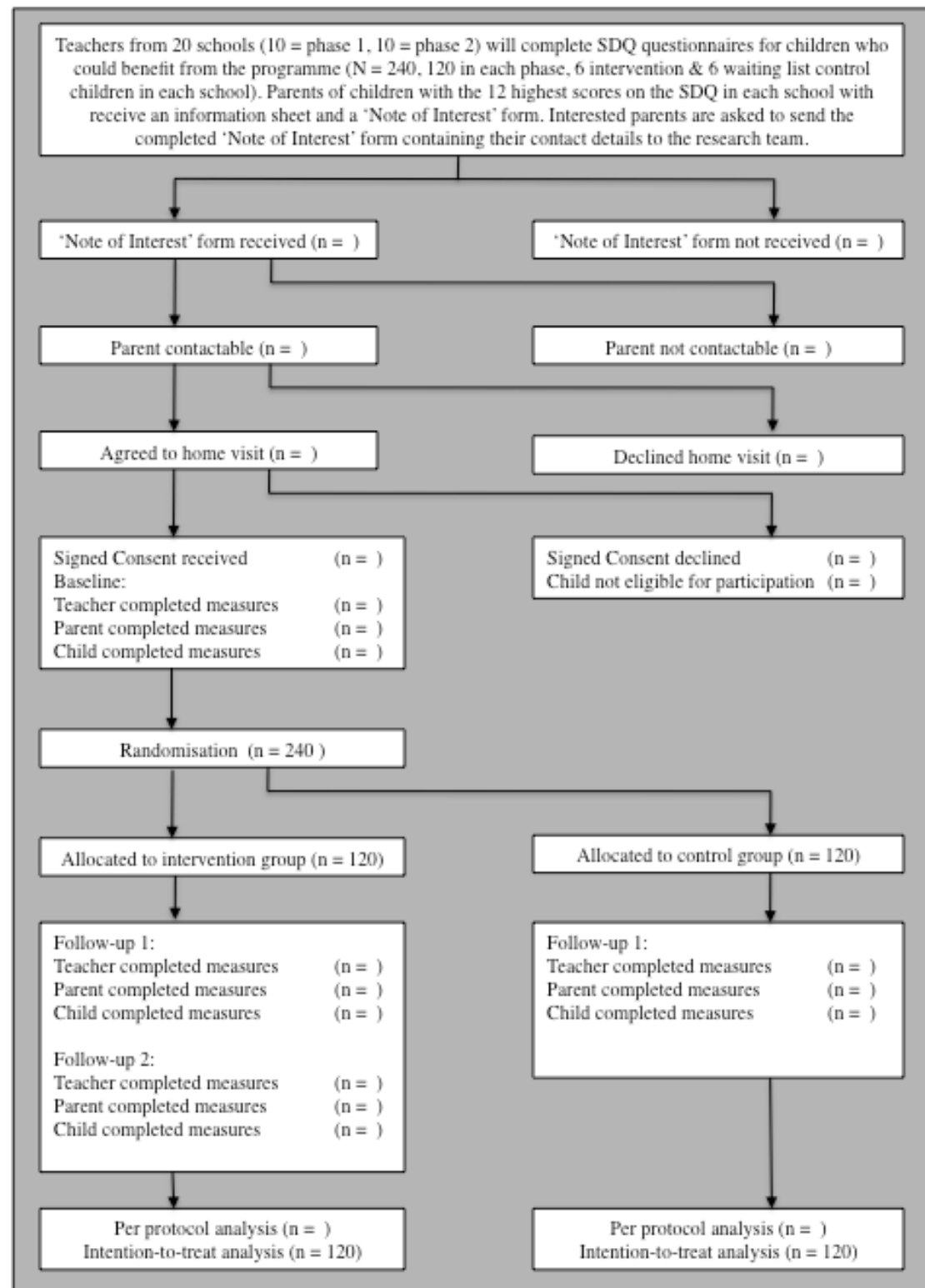

Supplement: Additional file 2 — Participant flow chart. A chart to show flow of participants through the trial. [file 1745-6215-12-39-S2.PDF]
